# The association between anesthesia method and perinatal outcomes in placental invasion anomalies

**DOI:** 10.12669/pjms.42.8.16082

**Published:** 2026-08

**Authors:** Seyda Kayhan Omeroglu, Ugur Uzun

**Affiliations:** 1 Seyda Kayhan Omeroglu, MD Department of Anesthesia and Reanimation, Dr. Suat Seren Chest Diseases and Surgery Training and Research Hospital, Izmir, Turkey; 2 Ugur Uzun Department of Anesthesia and Reanimation, University of Health Sciences Tepecik Training and Research Hospital, Izmir, Turkey

**Keywords:** Abnormal placental invasion, Maternal morbidity, Neonatal morbidity, Obstetric anesthesia, Peripartum hemorrhage

## Abstract

**Objectives:**

To evaluate the effects of different anesthesia procedures and surgical approaches on maternal and neonatal outcomes in cases of abnormal placental invasion (API), and to determine the factors that predict the transition from spinal anesthesia to general anesthesia.

**Methodology:**

This retrospective cohort study included 115 patients who underwent API surgery at University of Health Sciences Tepecik Training and Research Hospital, Izmir, Turkey; between 2015 to 2023. Demographic, obstetric, anesthetic, surgical, and neonatal data were analyzed. Comparisons were made based on the anesthesia type (spinal, general, and spinal-to-general conversion) and depth of invasion.

**Results:**

Placenta percreta was detected in 67% of the cases. As the severity of invasion increased, the operative time, blood and fluid replacement, and hospital stay were significantly prolonged (p<0.05). The American Society of Anesthesiologists (ASA) score, blood product consumption, and intraoperative complication rates were significantly higher in patients undergoing general anesthesia (p<0.001). Spinal anesthesia was the first choice (91.3%), and 47.6% of the cases required intraoperative conversion to general anesthesia. ROC analysis revealed that colloid use, ASA score, and fibrinogen requirement were significant predictors. There were no statistically significant differences in neonatal outcomes between the groups.

**Conclusion:**

In API cases, initiating neuraxial anesthesia, which allows for transition to general anesthesia when necessary, may improve maternal safety. Parameters such as colloid use and ASA score may guide early identification of high-risk cases. Prospective multicenter studies are required to establish standard management protocols for this high-risk group.

## INTRODUCTION

Abnormal placental invasion (API) is characterized by pathological invasion of the placenta beyond the endometrium during pregnancy, leading to uncontrolled massive bleeding at delivery and significantly increased maternal morbidity and mortality.^1^,^2^ Failure to effectively control bleeding can lead to serious complications such as intensive care admission, need for mechanical ventilation, secondary infections, and prolonged hospitalization.^3^–^5^ Therefore, in these high-risk cases, the surgical approach along with anesthesia management becomes a critical factor that directly affects the maternal and neonatal prognosis. 6

There are differing opinions in the literature regarding anesthetic management of API cases, and there is no standard approach accepted by all clinicians. It has been advocated that inhalational anesthetics may cause uterine relaxation, increasing intraoperative blood loss; therefore, the need for transfusion and starting the operation with neuraxial anesthesia is recommended. However, there are also data supporting the preference for general anesthesia to reduce the risk of intraoperative complications.^7^ Therefore, anesthesia management and surgical planning must be individualized for patient.^8^ The optimal anesthesia and surgical approach in these patients is still a matter of clinical debate because the cases are quite heterogeneous, and it is difficult to reach a consensus.

This study aimed to evaluate the anesthesia procedures of API cases together with their surgical approaches, retrospectively analyze the effects of these approaches on maternal and neonatal outcomes, and develop optimal management strategies to increase safety in this high-risk patient group based on the findings obtained.

## METHODOLOGY

This retrospective cohort study examined the electronic health records of 115 patients aged 18 years and older who underwent cesarean sections with a diagnosis of API at University of Health Sciences Tepecik Training and Research Hospital, Izmir, Turkey between November 2015 to October 2023. Seven patients with incomplete electronic records and one patient with a coagulation disorder were excluded from the study.

### Ethical Approval:

This study was approved by the Ethics Committee of the University of Health Sciences Tepecik Training and Research Hospital (Approval number: 2025/01-05, Dated: February 5, 2025).

Cases were included only in cases of histopathologically API (placenta accreta, increta, or percreta). Confirmation of pathological placenta accreta is the absence of the decidua basalis layer and the demonstration of placental villi entering the myometrium through this space. The variables examined in the study; demographic (age, body mass index (BMI)), obstetric characteristics (gravida, parity, abortion, dilation and curettage (D&C) history, number of previous cesarean sections, placenta previa, gestational age, prenatal diagnosis, mortality), comorbidities (hypertension, diabetes etc.), smoking, surgical factors (skin and uterine incision type, hysterectomy type, hypogastric artery ligation, operation time), anesthesia parameters The American Society of Anesthesiologists (ASA) score, type of anesthesia, hospital and intensive care unit (ICU) stay, maternal mortality, hematological data (preoperative and postoperative Hb, fibrinogen levels, presence of anemia, Hb change percentage), blood and fluid replacement (erythrocytes, fresh frozen plasma (FFP), platelets, fibrinogen, crystalloid, colloid, tranexamic acid) and intraoperative complications (bladder, ureter, bowel injuries), neonatal variables; birth weight, low birth weight (LBW) (<2500 g), APGAR scores (at 1st and 5th minutes), were grouped as neonatal respiratory distress syndrome (RDS), neonatal intensive care unit (NICU) admission and perinatal mortality.

### Statistical analyses:

Statistical analyses were performed using the IBM SPSS® 26 (IBM Corp. Released 2019. IBM SPSS Statistics for Windows, Version 26.0. Armonk, NY: IBM Corp) software. The conformity of the variables to a normal distribution was examined using analytical methods (Shapiro-Wilk test). Descriptive analyses are expressed as mean ± standard deviation for normally distributed variables. Descriptive statistics were performed using frequencies and percentages for categorical variables obtained from the sociodemographic and clinical information. One-way ANOVA and post hoc Bonferroni tests were used to compare continuous data types (demographic, clinical, obstetric, radiologic biochemistry, hormone, and hematologic laboratory results) between invasion and anesthesia types. Pearson’s chi-square test was used to compare categorical variables. Statistical significance was set at p < 0.05.

## RESULTS

In this study, 115 patients diagnosed with API were evaluated. The findings are presented in Table-I according to the type of API (accreta, increta, and percreta). The mean maternal age, BMI, parity, and comorbidities were similar between the groups (p>0.05). The presence of placenta previa and prenatal diagnosis were significantly higher in the Percreta group (p<0.05). In terms of skin incision type, infraumbilical midline incision was significantly more common in the Percreta cases (p < 0.001). The hysterectomy type also differed according to the severity of invasion, with the total hysterectomy rate being higher in the Percreta group (p = 0.010). Operative time was significantly longer (p = 0.001) and intraoperative crystalloid use was higher (p = 0.004). in the Percreta group.

**Table-I:** Comparison by API type.

Variables	Invasion			p
	Accreta (n=19)	Increta (n=19)	Percreta (n=77)	
Maternal age (year)	32.0±4.6	33.8±4.4	33.7±4.3	0.301
BMI	29.6±2.6	29.3±2.1	30.8±3.5	0.109
Gravity	3.8±1.9	3.8±1.2	3.9±1.3	0.902
Parity	1.9±0.9	2.3±0.9	2.3±0.9	0.213
Abortion	0.42±0.51	0.42±0.61	0.52±0.74	0.769
D&C	4(21.1)	4(21.1)	16(20.8)	0.999
Previous CS history	1.7±0.95	2.1±0.91	2.1±0.95	0.226
Placenta previa	10(52.6)	16(84.2)	75(97.4)	<0.001
HT	4(21.1)	7(36.8)	32(41.6)	0.254
DM	5(26.3)	7(36.8)	28(36.4)	0.697
Smoking	3(15.8)	7(36.8)	17(22.1)	0.273
Gestational age at delivery (week)	35.4±3.2	34.6±2.7	34.6±2.1	0.381
Prenatal diagnosis	9(47.4)	12(63.2)	59(76.6)	0.037
***Skin Incision (n,%)***				
Pfannenstiel	11(57.9)	8(42.1)	14(18.2)	<0.001
Infra-umbilical midline incisions	8(42.1)	11(57.9)	63(81.8)	
***Uterine Incision (n,%)***				
Kerr	3(15.8)	1(5.3)	0(0)	0.003
Vertical	16(84.2)	18(94.7)	77(100)	
***Hysterectomy Type (n,%)***				
Total	13(68.4)	12(63.2)	61(79.2)	0.010
Subtotal	6(31.6)	7(36.8)	16(20.8)	
Hypogastric Artery Ligation (n,%)	3(15.8)	5(26.3)	19(24.7)	0.680
ASA	2.1±0.9	2.5±0.7	2.5±0.8	0.156
***Type of anesthesia (n,%)***				
Spinal	10(52.6)	10(52.6)	35(45.5)	0.899
General	1(5.3)	1(5.3)	8(10.4)	
General + Spinal	8(42.1)	8(42.1)	34(44.2)	
Hospitalization Period (hour)	122.5±53.1	107.6±36.3	149.9±59.5	0.006
ICU Hospitalization Period (hour)	21.0±8.3	25.6±9.3	25.2±9.4	0.186
Maternal Death	0(0)	0(0)	0(0)	1.000
Operation time (minute)	108.6±37.7	142.6±48.3	157.8±55.3	0.001
Preop Hb (g/dl)	10.6±1.5	10.4±1.3	10.5±1.3	0.920
Preop Anemia Hb<11 g/dl	10(52.6)	12(63.2)	52(67.5)	0.475
Postop Hb (g/dl)	9.9±1.0	9.9±1.3	9.9±1.2	0.991
Postop Anemia Hb<11 g/dl	16(84.2)	16(84.2)	63(81.8)	0.951
Preop – Postop Hb % change	76.8±118.7	48.0±178.9	30.7±137.4	0.512
PreOp Fibrinogen	330.6±52.6	313.2±56.0	337.5±49.1	0.176
PostOp Fibrinogen	282.3±59.8	265.1±46.8	280.1±45.0	0.437
Erythrocyte Suspensions number (unit)	3.3±3.1	3.3±1.4	3.7±2.0	0.715
Fresh Frozen Plasma number (unit)	2.0±2.7	2.1±1.3	2.2±1.4	0.877
Platelet Suspensions number (unit)	0.21±0.42	0.16±0.38	0.29±0.51	0.531
Fibrinogen (g)	0.79±1.2	1.2±1.4	1.4±1.2	0.194
Colloid	368.4±326.7	289.5±346.2	506.5±417.5	0.066
Crystalloid	2052.6±524.3	1973.7±389.9	2311.7±437.1	0.004
Transamin	447.4±283.6	473.7±262.1	564.9±296.4	0.188
***Intraoperative Complications (n,%)***				
Bladder Injury	3(15.8)	1(5.3)	15(19.5)	0.510
Ureteral Injury	0(0)	0(0)	2(2.6)	
Intestinal Injury	0(0)	3(15.8)	10(13.0)	
Bladder+Ureteral	0(0)	0(0)	1(1.3)	

Independent t test used for continuous data and Pearson’s chi square test used for categorical variables. p<0.05 considered significant.

***Abbreviations:*** API: abnormal placental invasion BMI: body mass index; D&C: dilation and curettage; ASA: The American Society of Anesthesiologists score; CS: caesarean section; HT: hypertension; DM: diabetes mellitus ICU: intensive care unit; Hb: hemoglobin.

Comparisons of the findings according to the type of anesthesia are presented in Table-II. The ASA score was significantly higher in the general anesthesia group (p = 0.011). When evaluated in terms of blood product use, red blood cell suspension consumption was significantly higher in the general anesthesia group (p < 0.001). Similarly, FFP use was also found to be higher in the general anesthesia group (p = 0.006). In terms of intraoperative complications, the incidence of bladder injury was higher in patients undergoing general anesthesia (p = 0.045). Colloid use was also significantly higher in the general anesthesia group (p = 0.001).

**Table-II:** Comparison by type of anesthesia

Variables	Type of anesthesia			p
	General (n=10)	Spinal (n=55)	General+Spinal (n=50)	
Maternal age (year)	30.7±3.7	33.9±4.4	33.5±4.3	0.107
BMI	30.6±4.5	29.8±2.8	30.9±3.4	0.109
Gravity	4.0±0.9	3.7±1.2	4.1±1.6	0.298
Parity	2.7±1.1	2.2±0.9	2.2±0.9	0.236
Abortion	0.40±0.52	0.40±0.60	0.78±1.54	0.196
D&C	0(0)	12(21.8)	12(24.0)	0.227
Previous CS history	1.8±0.79	2.0±1.0	2.1±0.94	0.731
Placenta previa	8(80.0)	48(87.3)	45(90.0)	0.667
HT	6(60.0)	16(29.1)	21(42.0)	0.119
DM	4(40.0)	15(27.3)	21(42.0)	0.268
Smoking	4(40.0)	11(20.0)	12(24.0)	0.387
Gestational age at delivery (week)	34.2±2.9	34.6±2.6	34.9±2.1	0.608
Preanatally diagnosis	7(70.0)	34(61.8)	39(78.0)	0.198
***Skin Incision (n,%)***				
Pfannenstiel	3(30.0)	21(38.2)	9(18.0)	0.073
Infra-umbilical midline incisions	7(70.0)	34(61.8)	41(82.0)	
***Uterine Incision (n,%)***				
Kerr	0(0)	4(7.3)	0(0)	0.104
Vertical	10(100)	51(92.7)	50(100)	
***Hysterectomy Type (n,%)***				
Total	10(100)	36(65.5)	40(80.0)	0.060
Subtotal	0(0)	19(34.5)	10(20.0)	
Hypogastric Artery Ligation (n,%)	4(40.0)	11(20.0)	12(24.0)	0.387
ASA	3.0±0.8	2.2±0.9	2.5±0.7	0.011
***Type of anesthesia (n,%)***				
Accreta	1(10.0)	10(18.2)	8(16.0)	0.899
Increta	1(10.0)	10(18.2)	8(16.0)	
Percreta	8(80.0)	35(63.6)	34(68.0)	
Hospitalization Period (hour)	172.7±55.6	123.3±51.2	148.1±60.3	0.011
ICU Hospitalization Period (hour)	29.4±7.1	23.4±11.1	24.9±7.0	0.157
Maternal Death	0(0)	0(0)	0(0)	1.000
Operation time (minute)	173.4±48.2	135.7±51.8	154.6±56.2	0.056
Preop Hb (g/dl)	10.1±1.3	10.5±1.3	10.5±1.3	0.664
Preop Anemia Hb<11 g/dl	6(60.0)	33(60.2)	35(70.0)	0.540
Postop Hb (g/dl)	9.5±1.0	10.0±1.3	9.9±1.1	0.432
Postop Anemia Hb<11 g/dl	10(100.0)	44(80.0)	41(82.0)	0.304
Preop – Postop Hb % change	-21.4±85.7	58.0±120.1	35.6±167.6	0.361
PreOp Fibrinogen	345.9±60.8	327.9±48.3	334.5±52.7	0.553
PostOp Fibrinogen	287.7±57.4	279.0±48.4	274.9±46.1	0.726
Erythrocyte Suspensions number (unit)	5.9±3.4	3.0±1.9	3.7±1.8	<0.001
Fresh Frozen Plasma number (unit)	3.5±3.3	1.8±1.4	2.2±1.2	0.006
Platelet Suspensions number (unit)	0.30±0.48	0.18±0.43	0.32±0.51	0.315
Fibrinogen (g)	1.8±1.0	0.9±1.2	1.5±1.2	0.015
Colloid	600.0±316.2	309.1±340.1	570.0±428.7	0.001
Crystalloid	2400.6±394.4	2118.2±471.1	2280.7±453.6	0.083
Transamine	700.0±258.2	472.7±279.2	560.0±296.9	0.046
***Intraoperative Complications (n,%)***				
Bladder Injury	3(30.0)*	8(14.5)	8(16.0)	0.045
Ureteral Injury	0(0)	0(0)	2(4.0)	
Intestinal Injury	0(0)	7(12.7)*	6(12.0)*	
Bladder+Ureteral	1(10)	0(0)	1(1.3)	

Independent t test used for continuous data and Pearson’s chi square test used for categorical variables. p<0.05 considered significant.

***Abbreviations:*** BMI: body mass index; D&C: dilation and curettage; ASA: The American Society of Anesthesiologists score; CS: caesarean section; HT: hypertension; DM: diabetes mellitus ICU: intensive care unit; Hb: hemoglobin.

**Fig.1:**
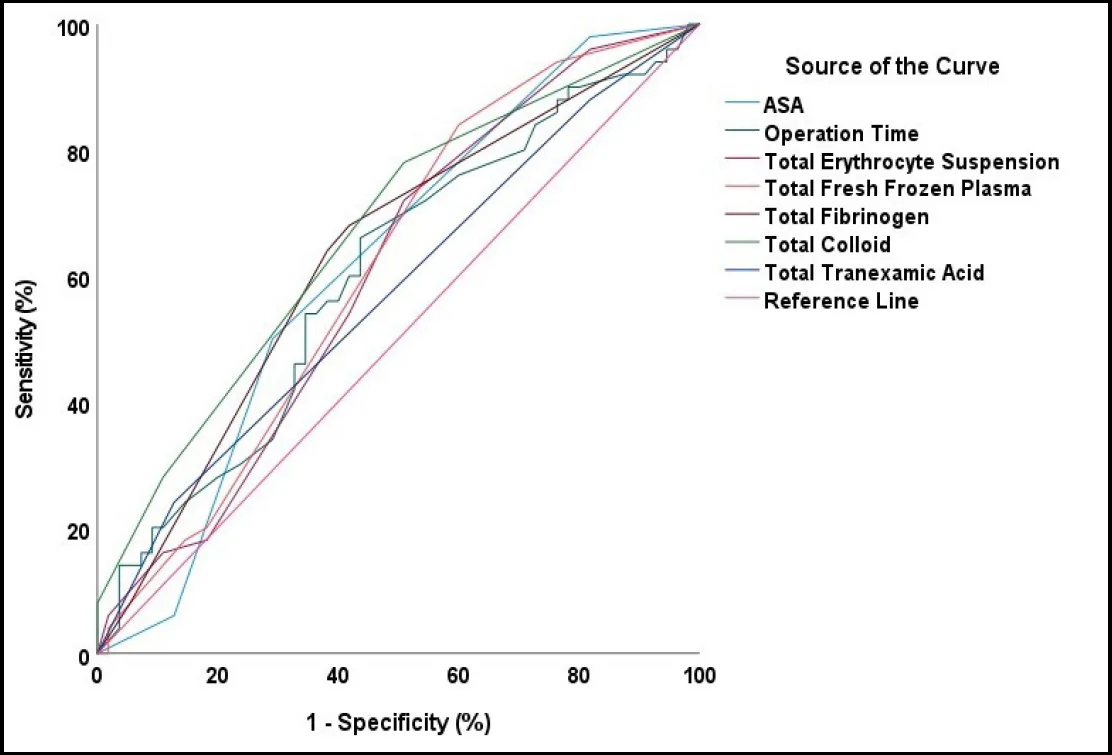
ROC and logistic regression analysis for predicting better parameter(s) to determine transition from spinal anaesthesia to spinal & general anaesthesia.

According to the ROC curve analysis of the parameters predicting the transition from spinal anesthesia to general anesthesia, ASA score (AUC = 0.62; 95% CI: 0.51–0.73; p = 0.035), fibrinogen use (AUC = 0.63; 95% CI: 0.53–0.74; p = 0.019), and colloid amount (AUC = 0.67; 95% CI: 0.57–0.77; p = 0.003) were determined as significant predictive variables (Fig.1). Cut-off points: 2.5 for ASA (sensitivity, 50%; specificity, 70.9%), 1.5 g for fibrinogen (sensitivity, 64%; specificity, 61.8%), and 250 mL for colloid (sensitivity, 78%; specificity, 50.9%). In logistic regression analysis, colloids were the only independent significant predictor (p = 0.020; OR = 1.001; 95% CI: 1.000–1.003). The overall model fit was statistically significant (χ²(4) = 14.18; p = 0.007) and the classification accuracy was 70.5% (Nagelkerke R² = 0.169).

The relationship between the type of anesthesia and neonatal outcomes is shown in Table-3. There were no significant differences between the type of anesthesia in terms of birth weight, low birth weight (LBW), APGAR scores, neonatal respiratory distress syndrome (RDS), admission to the neonatal intensive care unit (NICU), and perinatal mortality (p > 0.05).

**Table-III:** Neonatal outcomes according to anesthesia type.

Variables	Type of anesthesia			p
	General (n=10)	Spinal (n=55)	General+Spinal (n=50)	
Birth weight	2410.0±541.7	2543.8±502.0	2585.3±594.9	0.649
LBW (<2500 g)	5(50.0)	25(45.5)	23(46.0)	0.305
***APGAR Score***				
<7 at 1st minute	1(10.0)	10(18.2)	4(8.2)	0.305
<7 at 5th minute	1(10.0)	3(5.5)	0(0)	0.162
RDS in Newborns	9(90.0)	50(90.9)	43(86.0)	0.315
NICU Admission	9(90.0)	51(92.7)	48(96.0)	0.676
Perinatal Mortality	0(0)	0(0)	0(0)	1.000

One-Way ANOVA and Pearson’s chi square test used and p<0.05 considered significant.

Abbreviations: LBW: low birth weight ; RDS: respiratory distress syndrome ; NICU: neonatal intensive care unit

## DISCUSSION

In this study, our findings showed no difference in type of anesthesia according to invasion type; however, the rate of intraoperative bladder injury was up to 30% higher in cases treated with general anesthesia. Furthermore, high ASA scores and erythrocyte suspension (p < 0.001) and FFP (p = 0.006) rates were significantly higher in this group. These findings may be related to previous studies showing that inhalation anesthetics cause uterine relaxation, increasing intraoperative blood loss and thus the need for transfusions.^9^,^10^ However, studies have shown that regional anesthesia reduces blood loss by increasing uterine contractility through sympathetic blockade.^10^,^11^ In fact, in literature, it is recommended to start with neuraxial anesthesia in API cases and switch to general anesthesia when necessary.^12^

In cases of API, it has been stated that neuraxial anesthesia is appropriate during the cesarean section stage; however, when long-term surgery and high blood loss are expected after birth, switching to general anesthesia can be safe and often necessary.^13^,^14^ Literature reports that the most common reasons for conversion from spinal to general anesthesia include massive intraoperative hemorrhage, hemodynamic instability, inadequate spinal blockade, and prolonged surgical duration.^12^,^15^ Similarly, in our study, when patients who began spinal anesthesia and were converted to general anesthesia intraoperatively were examined, the operative time was longer in these patients, and the administered amounts of erythrocyte suspension, FFP, colloid, fibrinogen, and transamine were significantly higher. These findings support the idea that the primary reasons for conversion are the need for intraoperative bleeding management, hemodynamic instability, and a prolonged operative time. Furthermore, the higher ASA scores in patients who converted to general anesthesia suggest that the burden of systemic disease in these patients may be a determining factor in changing the type of anesthesia and the development of potential complications.

Intraoperative colloid use was the strongest predictor of conversion from spinal anesthesia to general anesthesia in our study. The ASA score and the amount of fibrinogen administered were also found to be significant predictors of this conversion. In patients with high ASA scores who began spinal anesthesia, increased intraoperative colloid use and decreased fibrinogen levels may be indicative of increased blood loss and transfusion requirements. This can be considered an important parameter that allows for the early identification of high-risk cases and the planning of proactive bleeding management strategies, enabling the prediction of conversion to general anesthesia.

The degree of placental invasion is a determinant of intraoperative blood loss and transfusion requirements.^16^ Clinical observations indicate that the use of red blood cell suspensions, fresh frozen plasma, platelets, fibrinogen concentrate, and tranexamic acid increases in these cases.^17^ Consistent with the literature, our study found significantly higher use of crystalloids and colloids (p = 0.004) in cases of placenta percreta. This emphasizes that, as the degree of placental invasion increases, intraoperative blood loss increases, and multidisciplinary hemostasis management is essential.^17^

When evaluated according to the type of anesthesia administered, it was observed that the entire uterine incision was performed vertically in both general and post-spinal general anesthesia cases, with Kerr incision being preferred only in the spinal anesthesia group at a rate of 7.3%. These findings demonstrate that surgical strategies are individualized not only based on the type of invasion but also on the clinical stability and anesthesia management of the case, and that clinical practice largely aligns with the recommendations in the IS-API guidelines. The rate of total hysterectomy increased significantly depending on the depth of invasion.^18^ The more frequent preference for a vertical uterine incision in this group is related to the lower uterine segment becoming less of a safe incision site due to invasion, facilitating easier placental separation, and preventing uncontrolled hemorrhage.^19^ A vertical incision allows safe fetal removal and facilitates the management of surgical complications.

Women with an Hb level below 90 g/L (<9 g/dL) at the time of delivery have an increased risk of bleeding during delivery and in the early postpartum period, regardless of the mode of delivery.^20^ The Hb level should be increased to the optimal level before surgery after the 28th week.^17^ The IS-AIP recommendation states that the hemoglobin level should be measured in women with an antenatal diagnosis of API, and if the Hb level is low (<110 g/L, i.e., 11 g/dL, before 28 weeks or <105 g/L, i.e., 10.5 g/dL, after 28 weeks), oral or IV iron therapy should be administered if necessary after appropriate hematological examination.^20^ In our study, the preoperative hemoglobin level of the patients was 10.46±1.34 g/dL, which decreased to 9.91±1.20 g/dL in the postoperative period. This decrease was most pronounced in patients undergoing general anesthesia, followed by those who were converted from spinal anesthesia to general anesthesia. The smallest decrease occurred in patients who received only spinal anesthesia. This suggests that initial regional anesthesia may reduce perioperative blood loss, even if general anesthesia is later converted.

Our findings also showed that increasing the invasion depth significantly prolonged operative and hospital stays, with the highest values observed in Percreta patients. This is consistent with findings in the literature that increasing invasion depth increases the risk of surgical difficulty, massive bleeding, and adjacent organ injury, thus prolonging the operative time.^17^,^21^ Furthermore, when evaluated according to the anesthesia type, the shortest hospital stay was observed in the spinal anesthesia group and the longest in the general anesthesia group, with the transition from spinal to general anesthesia occupying an intermediate position. The literature reports that general anesthesia prolongs the postoperative period due to increased blood loss and hemodynamic instability, whereas the transition from neuraxial anesthesia to general anesthesia is similar in intraoperative parameters and is more advantageous in terms of postoperative outcomes.^22^,^23^ Our study also observed that general anesthesia after spinal anesthesia offers better outcomes than general anesthesia alone. The lack of significant differences in intensive care unit stay suggests that this parameter may be due to multifactorial factors.

### Limitations:

The retrospective design of this study limits the establishment of causal relationships and increases the risk of selection and information bias. Furthermore, being a single-center study prevents the results from reflecting the diversity of practices and patient populations across centers, thus limiting the generalizability of the findings. The study sample size was limited, particularly in cases undergoing general anesthesia, which reduced the statistical power of the subgroup analyses. Additionally, uncontrolled variables, such as surgeon and anesthesia team experience and intraoperative decision-making processes, may have influenced the clinical outcomes. Another important limitation is the inability to monitor long-term maternal and neonatal outcomes, which prevents the assessment of late complications or effects on the quality of life. Finally, the lack of standardized anesthesia protocols at the time of the study led to heterogeneity among practices.

## CONCLUSION

Our study demonstrated the decisive impact of surgical and anesthetic management on maternal and neonatal prognosis in patients with API. Our findings indicate that, as the depth of invasion increases, surgical complexity, operative time, and the need for blood and fluid replacement significantly increase, which further reinforces the importance of a patient-specific multidisciplinary approach. The high blood product consumption and complication rates in patients undergoing general anesthesia suggest that initially opting for regional anesthesia followed by dynamic transitioning based on intraoperative conditions is more advantageous in terms of safety. Colloid use, ASA score, and fibrinogen requirement were significant predictors of the need for conversion from spinal to general anesthesia, and these parameters may be clinically valuable in the early identification of high-risk cases.

### Availability of data and materials:

The data were not published for ethical reasons. The data can be shared for the appropriate reasons. Further information can be provided by the corresponding author.

### Authors’ contributions:

**SKO:** Project development, data collection, and manuscript writing.

**UU:** data management, data analysis, and manuscript editing.

All authors have read and approved the final manuscript and are also accountable for the integrity of the study.
